# Items Too Numerous to Even Indicate in the Limited Space Allotted; All Interesting

**Published:** 1893-11

**Authors:** 


### Medical News and Miscellany.

**Dr. Finley Burts** has removed from Fort Worth to Dublin, Texas.

**Dr. Wm. Porter** has resigned the position of editor of the *St. Louis Clinique.*

**Died.**—Dr. G. W. McCollum, late of Georgia, died at Gainesville on October 17, aged 35.

**Dr. W. P. Burts** has again united with Drs. Field and Duringer in the practice under the old firm name of Burts, Field & Duringer, Fort Worth, Texas.

**Dr. T. J. Bennett,** editor of the *Texas Sanitarian,* is expected to return to Austin by the 15th inst., after a two months' attendance at the New York Polyclinic.

**The Texas Medical College** (Medical Department University of Texas) has a class of one hundred and nineteen matriculants this year, against twenty-four last year.

**Dr. A. J. Spencer,** one of Brazoria county's oldest and most esteemed citizens, died in San Antonio Saturday, October 14th, where he had gone for medical treatment.

**Attention, Doctors.**—If you want the latest and best operating chair at a reduced price for cash, or on easy payments, address, DRAKE & WOOD CO., Branch Office, Austin, Texas.

The homeopaths, according to *their* figures and diagnoses, *cure* more cases of diphtheria than all of the "old school" doctors put together; see? They can cure anything "cep'n death," as the little darkey said, and some of them can cure a mild case of that.

**By Proclamation** of the Governor, quarantine is raised on and after the 15th inst., as against vessels from "all ports south of 20° north latitude," and all the stations except Galveston will be closed on the 15th. The exception to this proclamation is, *vessels from known infected ports.*

**Wanted**—To exchange locations. A good residence and office in a thriving and beautiful little city, situated on a railroad in Central Texas, having superior school advantages, good society and churches. Will exchange for a good country location where there is no competition. Address Dr. J—., care TEXAS MEDICAL JOURNAL, Austin, Texas.

---

**The Unconscious Humorist.**—The *N. Y. Times*, a reformed homeopathic journal, calls the *Medical Century* (Fisher's journal —the homeopathic apostle)—"that unequalled depository of undesigned facetiæ."

Fisher is, himself, a good joke on mankind,—a kind of parody. "Undesigned," of course.

---

**The** JOURNAL invites contributions in the way of original papers on any subject connected with medicine or surgery, or reports of interesting and unusual cases. Few practicing physicians but see, every day, something which, if published, would be of interest and benefit to the brotherhood. Send along your observations, and give your brethren the benefit of your experience.

---

**The Texas Medical Journal** (Daniel's) for October is to hand, carefully noted. The JOURNAL, although red and fiery, is evidently improving with each added number, and is now a journal to be read and studied. It is a live affair, not to be thrown aside after a glance at its pages, but to be read, and reread. It is like the poles of a galvanic battery, when once you get hold you cannot let go.—*Lampasas Leader.*

---

**Died**—In Dallas, Texas, August 6. 1893, Mrs. Jennie Sutton, wife of Dr. W. H. Sutton, of that city. Mrs. Sutton was dearly beloved by all who had the happiness of knowing her. A Christian woman of large benevolence who did practical charity, she will be sadly missed by those to whose wants she ministered. A loving, devoted wife, and a tender mother—her household has been bereaved indeed. The JOURNAL extends its cordial sympathy to the mourning husband and children so sadly bereft.

---

**The Quarantine Station** on Chandeleur Islands was swept away in the terrible storm which devastated the coast on the 8th and 11th October, not a vestige of it being left except the foundation pillars of some buildings; no lives lost. The whole topography and contour of the island was changed, canals being cut clear through and across, rendering the island useless for quarantine occupancy in future. The station will be rebuilt on Ship Island and Surgeon Murray, M. H. S., is there now supervising the work of construction.

---

**Dr. J. M. Pratt,** one of the best known physicians residing in Hillsboro, was shot and killed near his home at Aquilla, twelve miles south of Hillsboro, about 8 o'clock on the night of Oct. 14, by W. C. Harris. There were no eye witnesses to the killing. At about 8:15 p. m., October 14, Harris drove up to the residence of Ben Goff, near the scene of the shooting, and informed him that he had killed Dr. Pratt and that in so doing he acted in self-defense. He then proceeded to Aquilla, where he wired Sheriff Bell at Hillsboro to come out and arrest him.

---

**To Solomon Solis:—**

"Now, I hold it's not the proper plan for any scientific gent,
to whale his fellow man;
Or if a member don't agree with his own peculiar whim,
to lay for that same member to put a head on him.
Nor is it ever proper for a scientific gent
to say another is an ass, at least to all intent;
Nor should the individual who happens to be meant,
reply by heaving rocks at him, at least to any great extent."

—*Bret Harte.*

---

**Bismuth Subgallate in Dyspepsia.**—Prof. Austin Flint (*N. Y. Medical Journal*) extols the subgallate of bismuth in ten-grain doses, as a most valuable remedy in functional dyspepsia. He gave the history of a case with flatulent distension of long standing, and producing great discomfort, in which he had run the gamut of salol mentholsalicylic acid, subnitrate bismuth, bicarbonate soda, and the list of pepsine preparations, without more than brief benefit, which was cured promptly by the newer remedy. Dr. Flint did not state whether the case was attended with diarrhœa or not, presumably it was. He has no confidence in the various pepsines and pepsine preparations.

---

**Minister of Public Health.**—At the annual meeting of the American Public Health Association, held at Chicago, October

9–14, 1893, the following resolution offered by Dr. Henry P. Walcott, of Massachusetts, was adopted:

*Resolved*, That the American Public Health Association again urge upon Congress the necessity of the appointment of some officer, with general sanitary authority, in connection with the national government.

That the functions of such an authority are of sufficient importance to demand the exclusive attention of the best instructed sanitarian.

That such authority should be enabled, from time to time, and under proper regulations, to secure the advice and co-operation of the state boards of health.

---

**Our Next Number** will be a Christmas edition, and will contain an extra amount of interesting matter. Advertisers should write for space in that edition while it may be had. *Verbum sat.*

The JOURNAL wishes to acknowledge the promptness and cheerful alacrity with which most of its subscribers responded to the "gentle reminder" of October 1, that it requires *some* money to run a stunning first-class A No. 1 journal, like the *Star* of *Texas*. Those who have not yet done so, have still an opportunity left them. We continue to take silver, postoffice orders, express orders, bank checks, or currency; anything to keep up a reputation for being clever fellows. Send along the remittances; Christmas is *a'most* here, and Betty and the baby expect every man to do his duty in this trying hour (trying to collect).

---

**An Accomplished Physician.**—The following comes from Oklahoma:

"Located at Perkins, and will visit Patient at ther home if so Desired.

"Dr. C. WHELTER and for Beast.

"Special attention will be taken in female complains, old or young, also in Midwifer at an call, and will treat cases of Rhumatism and Eplective fits, and the Doctor will keep on hand a salv that he makes himself, good for man, women alments warnted as good a salv that is in the united State for soorse or swelling saddle soores or collar Bruises, and will grow out a New hoof and the Doctor will make a syrup for coughs and cold and Plurise in the side or lungs charges Reasonable Consultation Free."—*Sanitarian.*

**The Texas Dysentery Cure.**—Since D. Knox's article on his case, cured by the ugly little thorny Mexico-Texas shrub, the JOURNAL has been permitted to see a letter to Dr. Knox from Dr. A. N. Perkins, the well-known veteran physician of Sabine Pass, in which Dr. Perkins says: "The preparation of the Mexican root, *Chapparo Amargosa*, you kindly sent me, was duly received, and I am happy to say it has cured my patient. I never saw a man improve so rapidly in his condition. He had utterly despaired of ever getting well, and his physician had despaired of ever curing him. He is now one of the happiest men in Texas. It is certainly a wonderful medicine. * * * I hope some energetic druggist will soon put a supply of it on the market, in the form of a fluid extract, for I repeat, it is a wonderful medicine. Please accept my sincere thanks for your kindness."

It was the *Texas Sanitarian*, we believe, that first called attention to this new-found and valuable drug.

---

**A Big Advance in Sanitary Science**—The Michigan State Board of Health adopted the following resolution September 30, 1893:

*Resolved*, That hereafter, consumption (and other diseases due to the bacillus tuberculosis) shall be included in the official list of "Diseases dangerous to the public health," referred to in sections 1675 and 1676 Howell's statutes, requiring notice by householders and physicians to the local health officer, as soon as such disease is recognized."

In a foot note, Henry B. Baker, the Secretary of the Michigan State Board says: "In this resolution the question of isolation of the patient is not mentioned. Its purpose is to secure to the local health authorities and to the State Board of Health information of the location of each case of this most dangerous disease, with the veiw of placing in the hands of the patient reliable information how to avoid giving the disease to others, and in the hands of those most endangered information how to avoid contracting this disease."

---

**Persistent Singultus Relieved by Lavage of the Stomach.**—Two interesting cases of persistent singultus have been reported recently by Gallant and Coleman, in which other remedies failing, immediate relief was obtained by washing out the stomach. The first patient, a man aged twenty-three years, after an attack of acute indigestion, began to hiccough on the evening of May

29. The paroxysms were frequent, and continued for the two following days, except during sleep. A saline, and the administration of small doses of cocaine, gave relief for two days, when the paroxysms began again, and continued until the afternoon of June 6, when the stomach was washed out with three quarts of warm water. The singultus at once ceased, and did not return.

Coleman's patient was seventy years old, and the hiccough had resisted household remedies for several days, and had been unrelieved by musk, bromides, chloral, or opium. Hiccoughing continued under profound narcotism, and when the patient was seen at the end of the ninth day, he was exhausted and oblivious of his surroundings. The stomach was washed out, the current bringing back no food, but a quantity of dark tenacious mucus, with patches of separated mucous membrane, very much thickened. The singultus ceased immediately, and the patient rapidly recovered.—*Boston Med. and Surg. Journal.*

---

**Punishment for the Crime of Producing Abortion.**—This crime has been particularly rampant in this city in the past year. A statement of the punishment accorded to it in various countries of Europe may therefore prove interesting. In England and Ireland the punishment is penal servitude for life, or a less term. Should the mother die, the crime becomes murder, which may be punished by death. In Scotland (says *The Lancet*) the punishment is arbitrary; in France, Spain, the German Empire, Austria, Hungary, Italy, Russia, Norway, Sweden, and Denmark—in short, throughout the whole of Europe—the crime is punished with imprisonment for from six months to twenty years, or for life. In Sweden the penaly is death if the mother dies; and in Russia the mother, if a consenting party, may be exiled to Siberia; in the Dominion of Canada the penalty is imprisonment for life; in Nova Scotia, Quebec, Ontario, British Columbia, and in Prince Edward Island it varies from imprisonment for two years to for life; in New Brunswick the penalty is death; in Australia and New Zealand the punishment is very severe, ranging from two years' imprisonment to penal servitude for life. In the United States it is punished with fines ranging from $100 to $5000, with imprisonment for long periods, and death.

There is as will be seen, a great unanimity of view regarding the high degree of criminality of the practice. Despite all this, the crime is increasing, both in our country and in Europe.

There is no doubt that this is due to the fact that many more women than formerly know of the possibility of abortion and refuse to accept the consequence of indiscretion or the responsibilities of maternity. There is but one way to look at it however—abortion involves the destruction of one life and danger to another. Hence it is a crime never to be justified even under extreme circumstances.

It is true that many obstetricians still regard the induction of premature labor, even before the infant is viable, as a necessary measure in certain very rare cases. But the indications for it are becoming more and more restricted as medical science advances, and it is not improbable that the time is near when the operation will no longer be thought of as a means of saving life. This legitimate obstetrical procedure, however, has nothing in common with criminal abortion, and medical men owe it to the community as well as to their profession to assist in limiting the work of those whose trade is infanticide. The daily papers could, doubtless, do more than any other agency toward abolishing the crime if they would consent to give up the small fees they get for advertising this class.—*New York Medical Record.*

And yet in the face of these facts, the *press*, which Mr. Rider-Taylor so cleverly defends in this issue, will, for a "small fee," publish advertisements of "Tansy Wafers," "Cotton Root Tea," "English Female Pills," and other abominable quack medicines *clearly intended* to produce abortion. They manage to keep just within the line which prohibits the mailing of immoral publications. Oh for a law to reach and punish those pure papers which our correspondent insists are not inimical to the medical profession, and which aid these quacks in the perpetration of a shocking crime—abortions by the score.

---

**Alumnol in Diseases of the Skin.**—Encouraged by their success in the discovery of dermatol and the continued reports of its value, Messrs. Heinz and Liebrecht have recently presented us with a new compound, which they have called alumnol. In its chemical composition it is a sulphonaphthalate of aluminium.

The discoverers set out with the idea of endeavoring to find an astringent which should be at the same time an antiseptic, and which should also be free from certain marked disadvantages which are present in almost all our available remedies of the class in question. These are almost all organic or inorganic salts of the heavy metals, and they form insoluble albuminates with the fluids of the tissues. This indissoluble coating confines the astringent and antiseptic action of these compounds within very narrow limits, and is the reason why their action is so very superficial.

Aluminium seemed to be the most promising of the lighter metals, and a long series of experiments were made in the attempt to find a compound of it that possessed the desired qualities. The organic salts of the metal, both of the fatty and the aromatic series, were systematically investigated, without any of them being found suitable. Then the double salts of tartaric acid with aluminium and the phenol compound were tried, but with the same result. At last the sulphonic-acid compounds were reached, and the desired combination was apparently obtained.

Heinz and Liebrecht have given us in alumnol a naphthoïsulphonic-acid salt of aluminium. It contains five per cent. of aluminium and fifteen per cent. of sulphur. It is a light, fine, white powder, non-hygroscopic and stable. It is soluble in water to the extent of forty-five per cent., and forms a permanent solution. It is freely soluble in glycerin. In alcohol it is only slightly soluble, and forms a blue fluorescent solution. It is soluble in ether. It strikes a blue color with ferric chloride, even in very small quantities. It gets darker on exposure to air, whether in solution, suspension, or in mixture. This, however, only renders it less sightly; it in no way interferes with its chemical or therapeutic properties.

Alumnol solutions are moderately acid, and they precipitate albumin and gelatin. But here at once we notice a marked and important difference between it and the other astringents that behave in the same way. The alumnol precipitate is soluble and redissolves in an excess of the albumin and gelatin, and the drug would thus theoretically possess penetrating and solvent powers far beyond those of the ordinary remedies of its class. In a fluid medium, such as pus, and especially where pus is compelled to flow out through narrow channels, it would seem to be especially valuable.

Heinz and Liebrecht have made an extended series of experiments to determine the properties of the new drug,* both in the

---

*H. Heinz and A. Liebrecht. Alumnol, a New Astringent and Antiseptic. Berl. kl. Woch., No. 46, 1892.

laboratory and in practice. Its antiseptic action was not found to be very marked. Cultures of *bacillus anthracis*, *micrcoccus prodigiosus*, etc., survived in it for over twenty-four hours. It did, however, prevent their further growth; 0.01 per cent. solutions hindered the development of the organisms of cholera, typhus, anthrax, pneumonia, and others, while 0.04 per cent. solutions stopped all further growth. The astringent action, on the other hand, was very marked. As dilute a solution as one of 0.0025 per cent. produced vascular contraction in the mesentery of the frog, and the reaction became very marked when strengths of 0.01 per cent. and over were reached. The exudation of the leucocytes in the vessels of the inflamed mesentery was very markedly diminished or completely stopped by the application of a solution of less than 0.01 per cent. Irritation was observed when the strengths of the solutions reached five per cent., but even with ten per cent. solutions no corrosive action was observed.

The diffusibility of the drug was shown by the following experiment. A quantity of a five per cent. nitrate of silver solution was injected into the right femoral muscles of a healthy rabbit, and the same quantity of an equally strong alumnol solution was injected into the left muscles. When the muscles were removed later, the site of the silver injection was found surrounded by a dense mass of coagulated albumin, and no silver could be found in the direct vicinity. The alumnol had apparently caused no irritation, and in the tissues for half an inch around the site of the puncture the alumnol-ferric-chloride reaction could be distinctly obtained.

As regards the action of the drug on the healthy animal and human organism, experiments on rabbits showed that strong solutions (ten to twenty per cent.) caused violent irritation and diarrhœa, but were not corrosive. Death occurred in a few rabbits, but only when the dose injected was very large, and frequently repeated. Even hypodermically it required seventy-three grains daily for a number of days in succession, to cause death. Sections showed that death was caused by disease of the kidneys, evidently due to the alnminium in alumnol. The doses requisite for these ill effects were so large, and the conditions needed so favorable, that there is no danger in its use in the human subject in any of the ordinary ways. In point of fact, Heinz and Liebrecht gave the drug internally to several hundred human subjects for long periods, and in larger doses. No unpleasant effect was observed, the urine was unchanged, and more especially contained no uluminium.

Therapeutically, the discoverers used alumnol in gynæcological, surgical, and dermatological cases for a period of more than twelve months. Its action as an astringent and antiseptic was very satisfactory, but it was in its use in diseases of the skin, and as an antagonist to the gonococcus, that it is of most interest to us. It was found useful in inflammation of the skin, both of the superficial and the chronic infiltrated varieties. Its miscibility with water, and the readiness with which ointments, varnishes, paints, and plasters, could be made with it, rendered it very desirable in these cases. In the chronic cases a ten to fifty per cent. preparation was employed; in the acute ones a two and a half to five per cent. one was found best.

Chotzen * has used it in over three hundred cases of skin and venereal diseases, and with the best results. He employed it pure, as a ten to twenty per cent. powder with talc and amylum, as a one to ten per cent. solution in water or alcohol, as a two and a half to ten per cent. lanoline salve, and as a varnish, with various proportions of collodion, traumaticin, etc. His results in the most varied skin diseases—in chancroid, urethritis, elytritis, etc.—were so good that it seemed desirable to make further experiments in the same direction. During the months of April and May, of this year, the remedy was employed in over sixty cases of dermatological, surgical, and venereal nature, in the German West Side Dispensary.

[Record of cases omitted, also experience with the drng in gonorrhea—Ed.]

The general results in sixteen cases of acute catarrhal inflammation of the skin were therefore very satisfactory. They were partly due, of course, to the protection afforded to the imflamed surface by the ointment and collodion covering, and partly, also, to the fact that most of the cases were in young individuals, and of short standing. Any occlusive dressing does good in these cases. But some of the cases, especially Nos. 6 and 7, were very obstinate ones, in which a great many other methods of treatment had been tried without avail. There were hardly any symptoms of irritation observed due to the preparations of the strength used; in fact, the very slight astringency seemed to

---

* Martin Chotzen. Alumnol, neues Mittel gegen Hautkrankheiten und Gonorrhœe. Berl. kl. Woch., No. 48, 1892.

have a soothing effect. I should consider alumnol as superior to zinc and bismuth in these cases, as being more uniform in results, and more generally applicable.—*William S. Gottheil, in New York Medical Journal.*

